# Nonmedical Uses of Antibiotics: Time to Restrict Their Use?

**DOI:** 10.1371/journal.pbio.1002266

**Published:** 2015-10-07

**Authors:** Richard William Meek, Hrushi Vyas, Laura Jane Violet Piddock

**Affiliations:** Institute of Microbiology and Infection, University Of Birmingham, Birmingham, United Kingdom

## Abstract

The global crisis of antibiotic resistance has reached a point where, if action is not taken, human medicine will enter a postantibiotic world and simple injuries could once again be life threatening. New antibiotics are needed urgently, but better use of existing agents is just as important. More appropriate use of antibiotics in medicine is vital, but the extensive use of antibiotics outside medical settings is often overlooked. Antibiotics are commonly used in animal husbandry, bee-keeping, fish farming and other forms of aquaculture, ethanol production, horticulture, antifouling paints, food preservation, and domestically. This provides multiple opportunities for the selection and spread of antibiotic-resistant bacteria. Given the current crisis, it is vital that the nonmedical use of antibiotics is critically examined and that any nonessential use halted.

## Abstract 2

Antibiotic resistance threatens the effective treatment of many bacterial infections. This Essay proposes that as any exposure to antibiotics promotes resistance, the extensive nonmedical use should be reduced to preserve this life-saving resource.

## Introduction

The crisis of antibiotic resistance compromises the ability to treat infections and threatens many areas of medicine, including surgery. This problem has been exacerbated by the drastic decline in the development of new antibiotics. Even with greater public awareness of the scale of this problem, only two new antibiotics with activity against infections for which there is most need, those by gram-negative bacteria, have been launched in the 12 months preceding March 2015, and these have limited indications. These two new drugs are combination agents where an inhibitor of a resistance mechanism (β-lactamase) is provided with a currently licensed antibiotic (ceftolozane/tazobactam and ceftazidime/avibactam). Most new antibiotics only target gram-positive bacteria.

The enormous threat posed by antimicrobial resistance has been recognised at national, regional, and global levels. In May 2014, WHO documented the global extent of antimicrobial resistance, reiterating the need for urgent action [1], while the World Health Assembly has emphasised the need to implement a global action plan [2]. Given the potential scale of the problem, the steps taken to combat antimicrobial resistance, particularly in terms of funding to implement action plans, have been inadequate [3].

## The Origins of Antibiotic Resistance

Antibacterial drug (hereafter referred to as “antibiotic”, as this term has been used synonymously in most recent publications) resistance is an illustration of natural selection. When bacteria are exposed to an antibiotic, those able to survive in its presence survive, proliferate, and spread. Resistance can arise de novo from spontaneous mutations in patients during antibiotic treatment (e.g., [4]), or in animals [5]. Often, resistance is spread by the sharing of antibiotic resistance genes on small mobile genetic elements (such as plasmids) that are readily transferred, even between distantly related bacterial species. Notably, nonpathogenic bacteria can pass on antibiotic resistance genes to pathogenic species present in the same environment.

**Fig 1:**
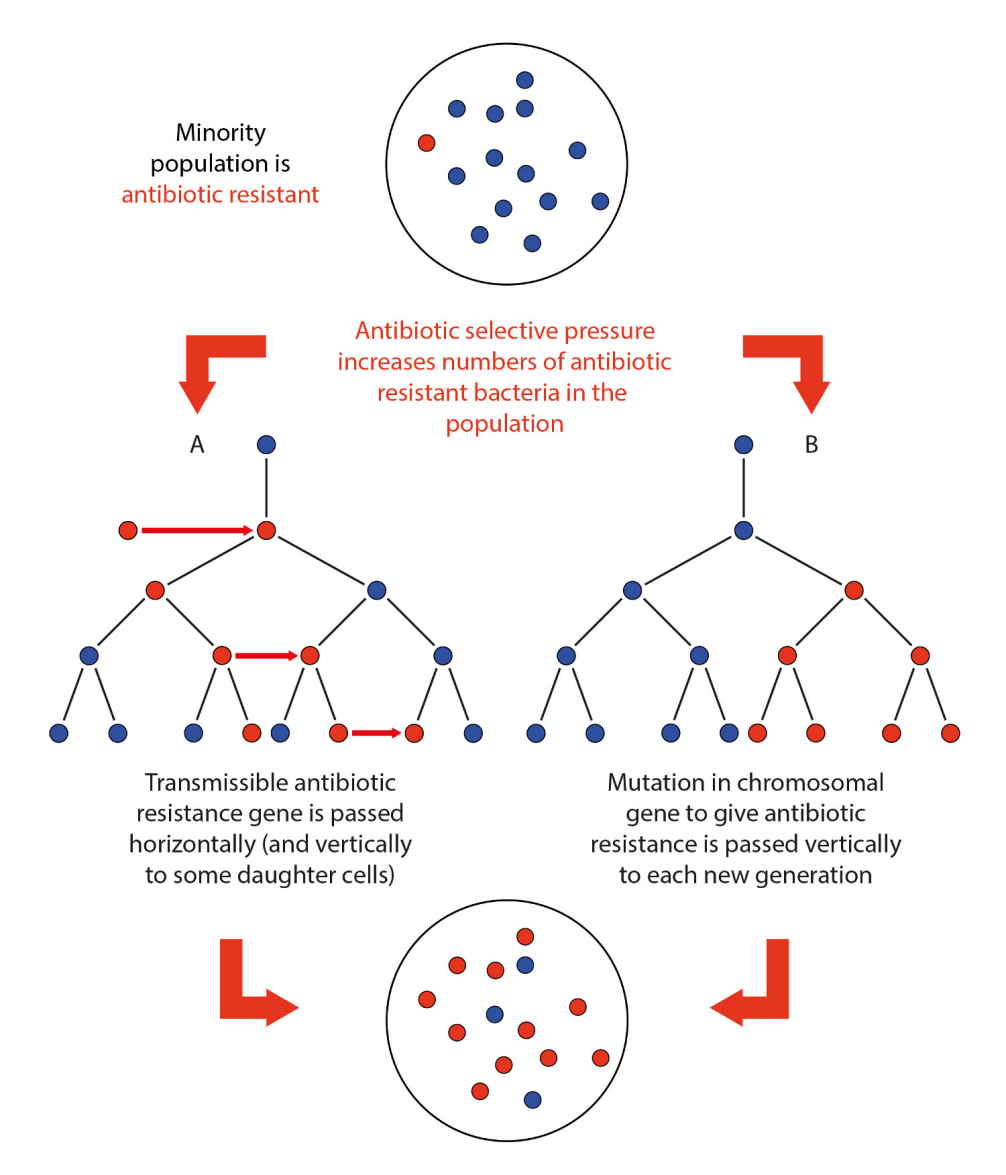
An illustration of how antibiotic resistance is selected in a bacterial population and how it proliferates. The large circle denotes an agar plate, test tube, human, animal, or other environment containing a population of bacteria. Small filled red circles, antibiotic-resistant bacterium. Small filled blue circles, antibiotic-susceptible bacterium. Red arrow denotes horizontal transmission between bacterial cells, black arrow indicates vertical transmission to daughter cells.

A crucial factor in the selection of antibiotic-resistant bacteria is the concentration of antibiotic that organisms are exposed to. The concentration of antibiotic that is required to inhibit the visible growth of bacteria is known as the “minimum inhibitory concentration” (MIC); the concentration required to kill bacteria is known as the “minimum bactericidal concentration” (MBC). The MIC and MBC values are higher for a resistant strain than for a susceptible strain. A concentration that is above the MIC for a susceptible strain but below that for a resistant strain allows resistant bacteria to multiply, while susceptible forms cannot (Fig 1). Furthermore, even at low concentrations of antibiotic, resistant strains are at an advantage, as their growth will be inhibited less than that of susceptible strains [6]. There is therefore a potentially large selective window of antibiotic concentrations favouring the growth of resistant bacteria, especially when resistance is at no fitness cost to the bacterium. Low-level exposure to some antibiotics is also associated with increased rates of mutation and horizontal gene transfer, increasing the likelihood that resistance will develop [7, 8].

Much attention has focused on overprescription and misuse of antibiotics in medical practice. Although the contribution that medical use has had to the antibiotic resistance burden cannot be overstated, this emphasis has overshadowed the widespread use of antibiotics outside human medicine. Resistance developed during nonmedical use of antibiotics can confer resistance to clinically useful antibiotics. Given the alarming depletion of the antibiotic arsenal, there is an urgent need to assess the extent and implications of antibiotic use outside medicine. Controversy has surrounded nonmedical uses of antibiotics due to a historical dearth of studies that directly correlate such use with the development of resistance and an impact upon human medicine. However, absence of evidence is not evidence of absence. This article outlines the available evidence that nonmedical use can select for antibiotic resistant bacteria.

## Use as Growth Promoters in Animals

Use of antibiotics in feed or water is used in some countries, e.g., the United States, to accelerate the growth and increase the size of animals bred for food. Remarkably, more antibiotics are used in the US for animal growth promotion than in human medicine. Dosing of antibiotics in growth promotion is not controlled leading to some animals receiving subinhibitory doses. This creates conditions that can favour the selection of resistant bacteria, which subsequently contaminate the environment.

Following the development of cross-resistance to antibiotics used in human medicine, several compounds were withdrawn from the European Union (EU) market. Use of growth-promoting antibiotics was further curtailed in 2006, with the implementation of an EU regulation banning the use of specific antibiotics of relevance to human health as animal growth promoters [8].

Arguments by industry for the continued use of antibiotics as growth promoters have focused on the potential economic impact of a ban and a possible effect on animal welfare [9]. Following concerns about the use of avoparcin and the emergence of resistance to a related important human antibiotic, vancomycin, as well as similar apprehensions with other antibiotics, Denmark discontinued use of all growth promoters in the 1990s. A 2002 WHO report suggested that the ban on antibiotic growth promoters in Denmark had only a small effect on pig production and no negative impact on poultry production [10]. Although use of antibiotics for veterinary purposes may have initially risen in Denmark, no such increases have been seen in nearby countries such as Norway, and in Sweden there was only a small increase before falling markedly [11]. Reduced use of growth promoters may have encouraged more attention on hygiene, vaccination, and nutritional intake to enhance yields.

In 1999, the US National Academy of Sciences calculated that a ban of antibiotic growth promoters would have minimal impact on the US livestock industry and consumers (adding US$4.84–9.72 to bills each year) [12].

## Veterinary Use

Antibiotics are also widely used in veterinary medicine to treat sick animals, to control the spread of disease, and to prevent infections. Few antibiotic classes are specific to human or veterinary medicine, so the emergence of resistance in one area has the potential to select resistant bacteria that infect man (and vice versa). Herds and flocks of animals are generally treated collectively, with antibiotics provided in food or water. This makes it very hard to control the dose, raising the risk that animals experience suboptimal levels of antibiotic and increasing the likelihood that antibiotic-resistant bacteria will be selected and cause infections in people that are difficult to treat. Of concern is that coupled with use for growth promotion, the use of antibiotics in animals is predicted to rise [13].

## Antibiotic Use in Aquaculture

Aquaculture (farming of fish and other marine life) is a huge global enterprise, particularly in Asia. Industrial scale farming is generally stressful to fish, impairing their immune systems and leaving them more vulnerable to infections. This has been used to justify widespread prophylactic use of antibiotics in aquaculture. Antibiotics are generally given as part of feed but may not be wholly consumed by the fish on a farm. Moreover, poor sanitary conditions encourage the growth of pathogenic bacteria, while high fish densities promote the rapid spread of antibiotic-resistant bacteria on a farm. Resistant microbes can also be rapidly disseminated through water to other farms and to wild animals [14]. As in animal husbandry, alternative measures such as less crowded pens, better sanitary conditions, and isolation of diseased fish could reduce the risk of infection and need for antibiotics.

Various other approaches could be used as alternatives to antibiotics in aquaculture, including vaccination or innovative methods such as phage therapy, probiotics, or disruption of signalling between bacteria to prevent biofilm formation. Vaccination is already widely used in countries such as Norway.

## Domestic Uses

In the home, numerous products are available to kill potentially harmful bacteria. Many such as soaps and shampoos contain triclosan, a synthetic compound with antibacterial, antifungal, and antiviral properties. As well as cleaning products, it has also been incorporated into solid products such as fabrics and children’s toys. Triclosan is active against many bacteria, but resistance can develop through a range of mechanisms. Notably, triclosan targets the bacterial FabI protein, and mutations in the corresponding *fabI* gene can confer triclosan resistance [15]. This also raises concerns about antibiotics targeting proteins similar to FabI such as the drug isoniazid used to treat TB. Furthermore, efflux pumps—proteins that eject antibiotics from the bacterial cell—also confer resistance to triclosan in a range of important microorganisms [16]. Development of triclosan-resistant bacteria therefore compromises the use of this product but also renders organisms less susceptible to a wide range of antibiotics that are used in human medicine.

Concerns have also been raised about the use of quaternary ammonium compounds (QACs), detergents, and antimicrobial chemicals used in a range of products, including facial cleansers, sun creams, mouthwashes, and sterilising agents for healthcare surfaces. QAC resistance genes are carried on mobile genetic elements that also harbour antibiotic resistance genes, so widespread use of QACs leads to the spread of both QAC and antibiotic resistance [17].

## Pollution of the Water Supply and Soils Are a Route for Antibiotic Resistant Bacteria to Transfer to Humans

Antibiotics may not be fully metabolised by the human body, and a large amount may be excreted in faeces or urine and enter wastewater treatment facilities. The slow rate of degradation of some antibiotics and use of treated and untreated sewage as fertiliser can also lead to the accumulation of antibiotics, and antibiotic-resistant bacteria, to levels that create selective pressures facilitating the spread of resistance.

Water systems are prone to the development of biofilms (slimy layers of communities of bacteria), which tend to be less susceptible to antibiotics [18]. Biofilms allow bacteria to persist in the presence of antibiotics, and water treatment may be less effective at killing bacteria in biofilms, again facilitating the spread of antibiotic-resistant organisms [19]. Although water treatments vastly reduce the number of bacteria in drinking water, it may favour the growth of antibiotic-resistant strains. Potentially, mechanisms that enable organisms to survive treatments such as chlorination could possibly enable them to withstand antibiotics. The numbers of several antibiotic-resistant bacteria have been found to be higher downstream of wastewater treatment plants, and resistance genes found in domestic and wild animals may reflect animals’ consumption of effluent water [20]. Antibiotic-resistant bacteria have also been identified in rivers in high income countries such as the United Kingdom [21]. These may originate from water treatment plants, sewage systems compromised by heavy rainfall, or run-off from farms. Water samples in cities such as New Delhi, India, have been found to contain important antibiotic resistance genes, probably as a result of contamination of water supplies by human faeces [22]. Such findings reinforce the need for clean water supplies and effective sewage systems to help tackle antibiotic resistance. Contamination of coastal waters provides another route for human exposure, directly via bathing or indirectly through consumption of contaminated shellfish.

Pharmaceutical production facilities are a further potential source of environmental contamination with antibiotics. In India, significant levels of antibiotics have been detected in wastewater treatment plants handling water from 90 pharmaceutical facilities—suggesting that there is a minimum of 45 kg of antibiotics being discharged every day [23]. Furthermore, human sewage was also found in this antibiotic-rich effluent, potentially exposing human pathogens directly to antibiotics.

Use of human or animal waste (manure) as a fertiliser on plants, which contains antibiotic residues or antibiotic-resistant bacteria is another possible route of dissemination of antibiotic-resistant bacteria or genes. Recently, culinary herbs exported from Southeast Asia have been found to harbour multidrug-resistant bacteria that are resistant to many antibiotics [24].

## Transmission of Antibiotic Resistance from Nonmedical Environments to Humans

Some sectors continue to dispute that the use of antibiotics outside of human medicine has any impact on medical practice. This has been because direct evidence of the complete sequence of events has been hard to demonstrate. This is for several reasons, not least the erroneous assumption that the identical strain of bacteria must be found, for instance, in both animals and humans [25]. There is increasing evidence that shows the selection of antibiotic-resistant bacteria due to nonmedical uses and transmission to humans. It is also clear that the events that lead to antibiotic resistance in human medicine are multifactorial, which careful molecular and large-scale genomic epidemiological analyses are helping to unravel [26].

Transfer of antibiotic-resistant bacteria to humans via animals is accumulating. Two examples are (1) antibiotic-resistant zoonoses selected after exposure to fluoroquinolone antibiotics in animals. For instance, fluoroquinolone-resistant *Campylobacter* spp. have been isolated from poultry [5], poultry products, and humans [27]. The chromosomally encoded resistance mechanism is identical, and the same strain types of Campylobacter have been isolated, irrespective of source. There is no evidence that selection of fluoroquinolone-resistant strains has occurred in humans as people are usually only treated with this antibiotic for chronic infections and the strains were isolated from samples taken prior to treatment. Infections with fluoroquinolone-resistant *Campylobacter* spp. can take longer to resolve. Therefore, until there is evidence to show that the large numbers of fluoroquinolone-resistant Campylobacter (from 15%–80% isolates depending on the country) isolated to date have not arisen during exposure of animals to these drugs, it is prudent to assume that the majority are of foodborne origin [2]. Pig farmers have a higher incidence of MRSA colonisation; this is attributed to strain exchanges between them and the pigs [28]. Recent studies have also indicated higher numbers of strains of livestock acquired MRSA strains than previously identified, and which were also isolated from infected people [29]. Novel resistance genes have also arisen in MRSA in cattle and have been subsequently isolated from milk and people, further suggesting transfer between these ecosystems [30]. Infections by MRSA can be difficult to treat, especially if in the bloodstream. Luckily, there are now several new antistaphylococcal antibiotics available and more in late stage development [31].

Transfer of antibiotic-resistance genes to strains of bacteria that colonise and infect humans, through pets (companion animals) or animals reared for food production, is also occurring. Two examples are (1) a high incidence of a particularly problematic antibiotic resistance gene encoding an extended-spectrum beta-lactamase (ESBL; giving resistance to penicillin-like drugs) has been found in bacteria isolated from humans and retail chicken meat [32]. The CTX-M-15 ESBL gene and surrounding genetic regions in human bacteria are identical to those found in *Kluyvera* spp. [33], an organism that occasionally infects people but is more often found in river water. It is postulated that these bacteria were ingested from contaminated water, and the *Kluyvera* spp. gene was mobilised and transferred to human commensal bacteria, which then caused an infection and/or transferred the resistance gene and element to human pathogenic gram-negative bacteria such as *Escherichia coli*. ESBL-producing gram-negative bacteria are a huge global healthcare burden and have caused the large increase in use of carbapenem antibiotics in human medicine. (2) There is evidence that antibiotic resistance genes arising in aquaculture have been transferred to bacteria that infect people. The human pathogen *Salmonella* Typhimurium DT104, for example, has been found to carry resistance genes that appear to have arisen in fish pathogens [34]. An *Aeromonas* plasmid may also have been transferred (with associated antibiotic resistance genes) to a range of human pathogenic bacteria, including *Salmonella enterica*, *Yersinia pestis* (the cause of plague), and *Vibrio cholerae* [35].

Whilst some would still consider the evidence above as speculative, it is unlikely that each event in the process will be categorically identified, because it would require the agreement of many different individuals to participate, including farmers (sometimes large corporations as in the US), veterinarians, abattoirs, food-preparation and packaging plants, healthy volunteers, and patients. Obtaining ethical approval for such studies in people would be difficult and the cost for such a large study would be enormous and so prohibitive.

## Conclusion

The discovery of antibiotics was one of the most influential moments in human history. Antibiotics have transformed human medicine, and their bacteriostatic and bactericidal powers have been exploited in multiple applications. Unfortunately, society now takes antibiotics for granted. Their widespread use has undermined the very features that have made them such powerful contributors to human health and well-being, and resistant bacteria can be isolated from numerous environments including bees, plants, and ethanol production (Table 1).

**Table 1:** Other nonmedical uses of antibiotics with evidence of or potential transfer of resistant bacteria or genes to humans and impact.

Use	Evidence	Potential or actual impact	Alternative to antibiotic use?
**Bee-keeping**			
Oxytetracycline to treat or prevent foulbrood, bacterial infections of bee larvae that can destroy entire bee colonies.	Increased MICs of oxytetracycline in bacteria isolated from bees in countries using these control measures [36].	Transmissible resistance genes in bee-infecting bacteria have also been found in bacteria isolated from humans and foodstuffs (e.g., cheese, meats). Resistance genes may spread via intermediate organisms [37].	In the EU, infected hives are generally sterilized and burned, reducing unnecessary use of antibiotics.
	Honeybees do not metabolise antibiotics, which may therefore be present at surprisingly high levels in honey [38].	Could lead to the inadvertent consumption of antibiotics by people and generate subtherapeutic levels of antibiotics in the human gut, which could select resistant bacteria.	
**Horticulture**			
Streptomycin, for example, was long used to treat fire blight, infection with *Erwinia amylovora* affecting apple and pear orchards.	Streptomycin-resistant *Erwinia* common in the US, also in Israel and New Zealand [39]. Evidence for gene transfer between *Erwinia* and animal pathogens, with a distinct genetic element found in *Erwinia* and in a pig-infecting strain of *E*.*coli* [40]. This genetic insert has also been found in the human pathogen *Campylobacter jejuni*, which can also infect pigs [41].	To avoid the potential for environmental contamination, the US Environmental Protection Agency banned the import of plant agricultural produce treated with gentamicin from Latin America.	None
**Food preservation**			
Bacteriocins (nisin) added to dairy products and canned food to inhibit growth of pathogens such as *Listeria monocytogenes*	*L*. *monocytogenes* can develop resistance to bacteriocins, possibly due to changes in its cell surface [42].	Some interest in the medical use of bacteriocins. However, there is no reason to assume that clinical use would not select for resistant strains.	None
**Ethanol production**			
To prevent bacterial contamination during the fermentation process.	The grains produced in ethanol production form a nourishing feed known as “dried distillers” grains with soluble (DDGS), which are fed to farm animals.	Although it has been claimed that antibiotics are rendered inactive by distilling practices, a 2008 FDA report [43] found evidence for antibiotics in DDGS.	None
**Prevent barnacle build-up on boat hulls**			
Tetracycline to prevent build-up of bacterial biofilms to which larger organisms such as barnacles attach.	Tetracycline antibiotics in antifouling paint [44].	Use of tetracycline in aquatic ecosystems has the potential to select for antibiotic-resistant biofilm-causing bacteria.	None

We now need to value antibiotics more highly and recognise that for the foreseeable future, these drugs are a finite and diminishing resource whose use must be carefully managed. While improved “antibiotic stewardship” in medical practice is essential, use of antibiotics outside human medicine needs to be rigorously examined and reduced to the barest minimum. We have produced a series of recommendations to address unnecessary use outside human medicine (Box 1). Implementation of these recommendations would promote more judicious use of antibiotics–essential if we are to continue benefiting from modern medical practices in the future. This article is based upon an 11,000-word report to the UK All Party Parliamentary Group on Antibiotics [45].

**Box 1. Recommendations for Measures to Reduce Nonmedical Uses of Antibiotics**

**Animal Husbandry and Aquaculture**

- A global ban on the use of all antibiotic growth promoters in animals.
- Increased monitoring of aquaculture and animal husbandry welfare practices to minimise the need for therapeutic drugs.
- Increased investment in research into minimising stress and disease in aquaculture and farm animals to limit the need for therapeutic drug use.
- Withdrawal of certain antibiotics from veterinary medicine, with specific classes of antibiotics reserved for human use.
- No use of any new classes of antibiotic in both animals and humans.
- Aquaculture facilities to be positioned distant from wild fish populations, to prevent dissemination of resistance genes.
- Increased research into diagnostics in animals and fish, to promote targeted treatment and prevent overuse of antibiotics.
- Increased infection prevention and control including the increased use of vaccination to prevent disease, with increased funding for new vaccine development.

**Bee-keeping**

- Global application of EU guidelines, limiting antibiotic use for treatment.

**Horticulture**

- A ban on antibiotic use in horticulture, with more research on containment systems and other means of control of plant pathogens.

**Food preservation**

- More research to assess whether bacteriocin use promotes cross-resistance to antibiotics used in human medicine, potentially followed by restricting their use to one area only.

**Alcohol production**

- Increased monitoring of antibiotic residues in alcohol production, with appropriate penalties when antibiotics are found.

**Boat and hull paint**

- Increased monitoring of antibiotic residues on boat hull surfaces, with appropriate penalties if residues are found.

**Domestic use**

- Withdrawal of triclosan from all domestic applications globally.
- Better disposal of triclosan waste.

**Water supply and pollution**

- Greater investment in sewage and water distribution systems, especially in low-income countries, to prevent antibiotic- and/or bacteria-contaminated water leeching into the environment.
- Development of improved methods to remove antibiotic residues, antibiotic-resistant bacteria, and antibiotic resistance genes from wastewater treatment plant effluent and drinking water sources.

## Abbreviations

DDGS: dried distillers grains with soluble
ESBL: extended-spectrum beta-lactamase
EU: European union
MBC: minimum bactericidal concentration
MIC: minimum inhibitory content
QAC: quaternary ammonium compounds
